# Blockade of vascular endothelial growth factor receptor 2 inhibits intraplaque haemorrhage by normalization of plaque neovessels

**DOI:** 10.1111/joim.12821

**Published:** 2018-09-07

**Authors:** M. R. de Vries, L. Parma, H. A. B. Peters, A. Schepers, J. F. Hamming, J. W. Jukema, M. J. T. H. Goumans, L. Guo, A. V. Finn, R. Virmani, C. K. Ozaki, P. H. A. Quax

**Affiliations:** ^1^ Department of Surgery Leiden University Medical Center Leiden The Netherlands; ^2^ Einthoven Laboratory for Experimental Vascular Medicine Leiden University Medical Center Leiden The Netherlands; ^3^ Department of Cardiology Leiden University Medical Center Leiden The Netherlands; ^4^ Department of Cell and Chemical Biology Leiden University Medical Center Leiden The Netherlands; ^5^ CVPath Institute Inc. Gaithersburg MD USA; ^6^ Department of Surgery Division of Vascular and Endovascular Surgery Brigham and Women's Hospital Harvard Medical School Boston MA USA

**Keywords:** angiogenesis, atherosclerosis, intraplaque haemorrhage, vascular endothelial growth factor, vein graft

## Abstract

**Background:**

Plaque angiogenesis is associated with atherosclerotic lesion growth, plaque instability and negative clinical outcome. Plaque angiogenesis is a natural occurring process to fulfil the increasing demand of oxygen and nourishment of the vessel wall. However, inadequate formed, immature plaque neovessels are leaky and cause intraplaque haemorrhage.

**Objective:**

Blockade of VEGFR2 normalizes the unbridled process of plaque neovessel formation and induces maturation of nascent vessels resulting in prevention of intraplaque haemorrhage and influx of inflammatory cells into the plaque and subsequently increases plaque stability.

**Methods and Results:**

In human carotid and vein graft atherosclerotic lesions, leaky plaque neovessels and intraplaque haemorrhage co‐localize with VEGF/VEGFR2 and angiopoietins. Using hypercholesterolaemic ApoE3*Leiden mice that received a donor caval vein interposition in the carotid artery, we demonstrate that atherosclerotic vein graft lesions at t28 are associated with hypoxia, Hif1α and Sdf1 up‐regulation. Local VEGF administration results in increased plaque angiogenesis. VEGFR2 blockade in this model results in a significant 44% decrease in intraplaque haemorrhage and 80% less extravasated erythrocytes compared to controls. VEGFR2 blockade *in vivo* results in a 32% of reduction in vein graft size and more stable lesions with significantly reduced macrophage content (30%), and increased collagen (54%) and smooth muscle cell content (123%). Significant decreased VEGF, angiopoietin‐2 and increased Connexin 40 expression levels demonstrate increased plaque neovessel maturation in the vein grafts. VEGFR2 blockade in an aortic ring assay showed increased pericyte coverage of the capillary sprouts.

**Conclusion:**

Inhibition of intraplaque haemorrhage by controlling neovessels maturation holds promise to improve plaque stability.

## Introduction

Plaque angiogenesis and intraplaque haemorrhage are critical determinants of plaque instability 1. Plaque angiogenesis or neovessel formation correlates with lesion progression, plaque inflammation and negative clinical outcome after cardiovascular events 2, 3. Fragile atherosclerotic plaques do not only cause plaque instability in native atherosclerosis but also in postinterventional lesions such as in vein grafts and in in‐stent neoatherosclerosis 4, 5.

Hypoxia in atherosclerotic lesions is a driver of plaque instability 6. Furthermore, it can induce lesion growth and affect vascular remodelling 7, 8. Angiogenesis, a natural occurring process induced by hypoxia, fulfils the increasing demand of oxygen and nourishment of the vessel wall. Neovessel formation is stimulated by hypoxia‐induced up‐regulation of vascular endothelial growth factor (VEGF) 9, 10. VEGF binds to and mediates its activity primarily through VEGF receptor 2 (VEGFR2). Plaque neovessels are frequently found dysfunctional, especially immature plaque neovessels. These neovessels are characterized by increased permeability caused by underdeveloped interendothelial junctions, incomplete basement membranes and partial pericyte coverage 11. As a result, neovessels leak blood components into the lesions, that is intraplaque haemorrhage. Erythrocytes in the plaque become phagocytosed, and their cholesterol‐rich membranes contribute to the free cholesterol content of the plaque 12, 13, 14. Leaky neovessels are clearly associated with inflammatory cells 1. Recently, it was shown by some of the coauthors that especially haemoglobin–haptoglobin receptor CD163+ macrophages interact with plaque neovessels and induce vascular permeability resulting in the propagation of the instable character of lesions 15.

Anti‐angiogenic therapies are used in cancer and eye diseases. However, these therapies are not always found beneficial 16. Normalization of the neovasculature, that is creating healthy mature neovessels, is a relatively new strategy to target neovascularization 17. Generation of a basement membrane and recruitment of pericytes are crucial steps in vessel maturation. These processes are regulated by VEGF‐VEGFR2 and the tightly balanced angiopoietin‐Tie2 system 18. High levels of VEGF increase vessel permeability, whereas low levels of VEGF are necessary for a stable vessel 19. Angiopoietin (Ang)‐1 mediates pericyte–endothelial cell adhesion, and Ang‐2 induces vessel permeability and acts as an antagonist to Ang‐1, resulting in pericyte loss 19.

In preclinical models, it has been demonstrated that pro‐angiogenic strategies augment atherosclerotic plaque growth and vascular inflammation, whereas anti‐angiogenic strategies inhibit atherosclerosis 20, 21, 22. Previously, we have shown that lesions induced by vein grafting in atherosclerosis‐prone mice display profound plaque neovessels and intraplaque haemorrhage 23. These plaque neovessels frequently lack pericyte coverage classifying them as immature 23.

We hypothesized that improving the maturation state of plaque neovessels reduces the extent of vascular ‘leakiness', which results in reduced intraplaque haemorrhage and lesion progression. Since low levels of VEGF are necessary for vessel homeostasis, we investigated the impact of the VEGFR2‐blocking antibody (DC101) on plaque angiogenesis, maturation status, and atherosclerotic lesion size and composition in murine vein grafts.

## Materials and methods

### Human tissue specimens

Human coronary artery vein graft specimens (*n* = 12) were available from the CVPath Institute. A detailed patient description can be found in Table S1. The severity of the vein graft lesions was scored as early, intermediate or late as described previously 4. Anonymous carotid endarterectomy (*n* = 12) specimens obtained at the LUMC in accordance with guidelines set out by the ‘Code for Proper Secondary Use of Human Tissue' of the Dutch Federation of Biomedical Scientific Societies (Federa) and conform with the principles outlined in the Declaration of Helsinki. The carotid endarterectomy specimen phenotype was scored based on the Athero Express Biobank classification 2. Unstable plaques were selected based on relative necrotic core size, foam cell and inflammatory cell infiltration score, and the presence of neovascularization. Specimens were formalin fixed, embedded in paraffin, sectioned and stained as described below.

### Animals

All animal experiments were performed in compliance with Dutch government guidelines and the Directive 2010/63/EU of the European Parliament. Male ApoE3*Leiden mice, crossbred in our own colony on a C57BL/6 background for at least 18 generations, 10–16 weeks old, were fed a diet (AB diets) containing 1% cholesterol and 0.05% cholate (VEGF experiment) or 0.5% cholate (time courses and DC101 experiment) from 3 weeks prior to surgery until sacrifice. The mice were housed on regular bedding and nesting material; water and diet were provided at libitum. Mice were randomized based on their plasma cholesterol levels (inclusion criteria of cholesterol level > 8 mol L^−1^; kit 1489437; Roche Diagnostics, Basel, Switzerland) and body weight. Mice were anesthetized with midazolam (5 mg kg^−1^; Roche Diagnostics), medetomidine (0.5 mg kg^−1^; Orion, Espoo, Finland) and fentanyl (0.05 mg kg^−1^; Janssen Pharmaceutical, Beerse, Belgium). After the surgery, the anaesthesia of the mice was antagonized with atipamezol (2.5 mg kg^−1^, Orion) and fluminasenil (0.5 mg kg^−1^; Fresenius Kabi, Bad Homburg vor der Höhe, Germany). Buprenorphine (0.1 mg kg^−1^; MSD Animal Health, Keniworth, NJ, USA) was given after surgery to relieve pain.

### Vein grafts

Vein graft surgery was performed by a donor caval vein interposition in the carotid artery of recipient mice as described before 23, 24. At sacrifice, patency of the vein grafts was visually checked for pulsations and blood flow, and occluded vein grafts were excluded from the study. Animals underwent 3 minutes of *in vivo* perfusion fixation with PBS and formalin under anaesthesia. Vein grafts were harvested, formalin fixed, dehydrated and paraffin‐embedded for histology.

### Treatment

VEGF experiment: Immediately after vein graft surgery, the vein graft was immersed *in vivo* in 100 μL of 40% pluronic gel (F127; Sigma‐Aldrich, St Louis, MO, USA) containing 250 ng VEGF (*n* = 7; Sigma‐Aldrich) or pluronic gel alone (*n* = 6).

DC101 experiment: Mice were treated with IP injections of rat anti‐mouse VEGF‐R2 IgG monoclonal blocking antibodies (10 mg kg^−1^ DC101; Bio X cell, Lebanon, NH, USA) 25 (*n* = 14) or control rat anti‐mouse IgG antibodies (10 mg kg^−1^; *n* = 14; Bio X cell) at days 14, 17, 21 and 25. Two mice in this group were excluded from analysis due to thrombosis in the vein graft.

### *In vivo* detection of hypoxia

One hour prior to sacrifice mice (*n* = 6) received an intraperitoneal injection with the hypoxia marker pimonidazole hydrochloride (100 mg kg^−1^; hypoxyprobe Omni kit; Hypoxyprobe Inc., Burlington, MA, USA). Pimonidazole was detected with the polyclonal antibody (clone 2627) that is included in the kit.

### Histological and immunohistochemical assessment of vein grafts

Cross sections were routinely stained with haematoxylin–phloxine–saffron (HPS) or Movat's pentachrome staining. Picrosirius red was used to detect collagen. The following antibodies were used for immunohistochemistry: endothelial cell CD31 (sc‐1506‐r; Santa Cruz, Dallas, TX, USA), Glycophorin A (YTH89.1; Thermofisher, Waltham, MA, USA), VEGF (sc‐7269; Santa Cruz), VEGFR2 (55B11; Cell Signalling, Danvers, MA, USA), Ang‐1 (human; A78648; Atlas antibodies, Bromma, Sweden; murine LS‐B62; LS Bio, Seattle, WA, USA), Ang‐2 (PAB19784; Abnova, Taipei, Taiwan), intercellular adhesion molecule 1 (ICAM1 sc‐1511‐r; Santa Cruz), vascular cell adhesion protein 1 (VCAM1; ab27560; Abcam, Cambridge, UK), stromal cell‐derived factor 1 (SDF‐1; ab9797; Abcam), hypoxia‐inducible factor 1‐alpha (HIF‐1α; NB100‐473; Novus Biologicals, Littletown, CO, USA), CD163 (orb13303; Biorbyt, Cambridge, UK), CD3 (ab16669; Abcam), macrophage MAC3 (550292; BD‐Pharmingen, Franklin Lakes, NJ, USA), smooth muscle cell actin (SMCA; 1A4, Dako, Santa Clara, CA, USA) and erythrocyte Ly76 (TER119; 116202; Biolegend, San Diego, CA, USA). For each antibody, isotype‐matched antibodies were used as negative controls.

Images of the human lesions were obtained with the Ultrafast Digital Pathology Slide Scanner and associated software (Phillips, Eindhoven, the Netherlands). Bright‐field photographs were obtained with a Zeiss microscope and associated software. Fluorescent double and triple staining were acquired with the fluorescent slide scanner (3DHistech, Budapest, Hungary) and panoramic viewer software (3DHistech).

### Morphometric analysis of vein grafts

Image analysis software (Qwin, Leica, Wetzlar, Germany) was used for morphometric analysis. For each mouse, eight (150 μm spaced) cross sections were used to determine lesion size and occurrence of intraplaque haemorrhage over a total vein graft length of 1050 μm. Since elastic laminas are nonexistent in these venous grafts, we analysed the putative vessel wall area (or lesion area) by measuring total vessel area (area within the adventitia) and the lumen area. The lesion area was calculated as total vessel area minus lumen area. Immuno‐positive areas in vein grafts are expressed as total area or percentage of the lesion area.

### Morphologic analyses of intraplaque haemorrhage

Intraplaque haemorrhage was analysed using CD31/Ly76 double‐stained sections. Lesions where erythrocytes were found extravascular, adjacent to neovessels, were regarded as lesions with intraplaque haemorrhage. Using image analysis software (Qwin, Leica), the extravasated erythrocyte content was evaluated by measuring the total erythrocyte area in the lesion, followed by subtraction of the area of erythrocytes within the CD31‐stained neovessels.

### RNA isolation, cDNA synthesis and RT‐PCR

Time course: Total RNA was isolated from murine vein grafts harvested at several time‐points [vein grafts/time‐point; t0 (caval vein); 24 h; 3 days; 7 days; (*n* = 3 each), 14 days (*n* = 4) and 28 days (*n* = 5)]. RNA was isolated, and cDNA was synthesized as described previously 26.

VEGFR2 experiment: Total RNA was isolated from 10 (20‐μm‐thick) paraffin sections of vein grafts (*n* = 6/group). RNA was isolated according to manufacturers protocol (FFPE RNA isolation kit; Qiagen, Venlo, the Netherlands). RNA for q‐PCR was reverse transcribed using a High Capacity RNA‐to‐cDNA kit (Applied Biosystems). Commercially available TaqMan gene expression assays for the housekeeping gene hypoxanthine phosphoribosyltransferase (HPRT1) and selected genes were used (Applied Biosystems, Foster City, CA, USA); *Vegfa* (Mm 00437306_m1), *Hif1‐*α (Mm 0468869_m1), *Sdf‐1 (Mm 00445553_m1), Vegfr2* (Mm01222421_m1), *Vegfr1* (Mm00438980_m1), *Tie2* (Mm00443243_m1), *Icam1* (Mm00516023_m1), *Ang‐1* (Mm00456503_m1), *Ang‐2* (Mm00545822_m1), *Connexin37* (Mm01179783_m1), *Connexin40* (Mm01265686_m1), *Connexin43* (Mm00439105_m1), *Ccl2* (Mm00441242_m1) and *Il6* (Mm00441242_m1)). q‐PCR products were performed on the ABI 7500 Fast system (Applied Biosystems). The 2‐ΔΔCt method was used to analyse the relative changes in gene expression.

### Aortic ring assay

Three separate experiments were conducted using three mice per experiment. C57BL/6 mice, age between 8 and 12 weeks, were anesthetized (as described above), and the aorta was dissected and stored in the medium. Each aorta was cut in 1‐mm rings and serum‐starved in Opti‐MEM + Glutamax (Gibco, Gaithersburg, MD, USA) overnight at 37 °C and 5% CO_2_. The next day, each ring was mounted in a well of a 96‐well plate in 70 μL of 1.0 mg mL^−1^ acid‐solubilized rat tail collagen I (Millipore, Burlington, MA, USA) in DMEM. After collagen polymerization (60 min at 37 °C and 5% CO_2_), Opti‐MEM supplemented with 2.5% FCS and 30 ng mL^−1^ VEGF (R&D systems, Minneapolis, MI, USA) was added with or without DC101 or control antibodies (30 μg mL^−1^). The rings were cultured for 7 days, and pictures were taken (Zeiss, Oberkochen, Germany). The number of sprouts was counted manually.

For immunohistochemistry, rings were formalin fixed and permeabilized with 0.2% Triton X‐100. Rings were stained with SMCA, CD31 (BD‐Pharmingen) and MAC3. Z stack images were captured with a LSM700 confocal laser‐scanning microscope (Zeiss) and quantified with ImageJ (Bethesda, MD, USA).

### Statistical analysis

Results are expressed as mean ± SEM. A two‐tailed Student's *t*‐test was used to compare individual groups. Non‐Gaussian distributed data were analysed using a Mann–Whitney *U*‐test using GraphPad Prism version 6.00 for Windows (GraphPad Software, La Jolla, CA, USA). Probability values < 0.05 were regarded as significant.

## Results

### Leaky neovessels in human vein graft and carotid lesions

**Figure 1:**
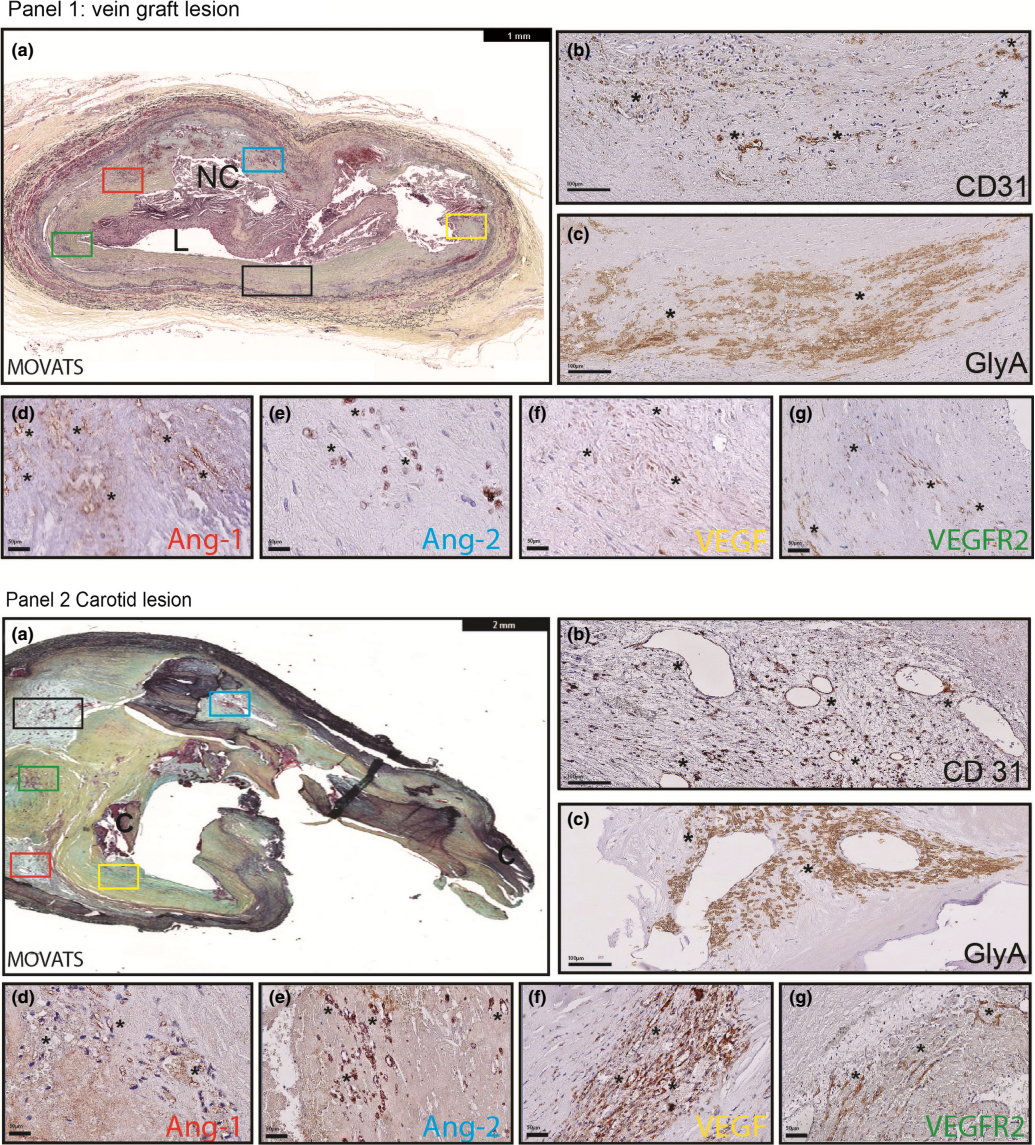
Leaky neovessels in human vein graft and carotid lesions. (a) Human vein grafts (*n* = 12) and (b) carotid plaques (*n* = 12) were stained with Movat's pentachrome for general morphology. (a/b.1) CD31‐positive leaky neovessels as revealed by the presence of (a.2/b.2) glycophorin A (GLyA)‐positive erythrocytes outside neovessels. (a.3‐4, b.3‐4) Angiopoietins, Ang‐1 and Ang‐2, localized around the neovessels as well as VEGF and VEGFR2 (a.5‐6, b.5‐6). NC, necrotic core. L, Lumen C, Calcification. *neovessels.

Both vein graft specimens (Fig. 1, panel 1) and carotid atherosclerotic lesions (Fig. 1, panel 2) show features of classical atherosclerotic lesions with, foam cells, calcification and necrotic cores. Neovessels were found throughout the lesions in both vein grafts and carotid specimen, with a preference for the media and at inflammatory regions around necrotic cores, Fig. 1(b) panels 1 and 2. Frequently, these neovessels were leaky as demonstrated by the presence of erythrocytes (Glycophorin A‐expressing cells) outside the neovessels, Fig. 1(c) panels 1 and 2. Both Ang‐1 [Fig. 1(d) panels 1 and 2] and Ang‐2 [Fig. 1(e) panel 1 and 2] were localized around the neovessels, although not all neovessels were found positive. Most neovessels, also in regions of intraplaque haemorrhage, did express VEGF, Fig. 1(f) panel 1 and 2. VEGFR2 staining was present around the neovessels but not as strong as VEGF expression, Fig. 1(g) panels 1 and 2.

### Hypoxia drives plaque angiogenesis in vein grafts

**Figure 2:**
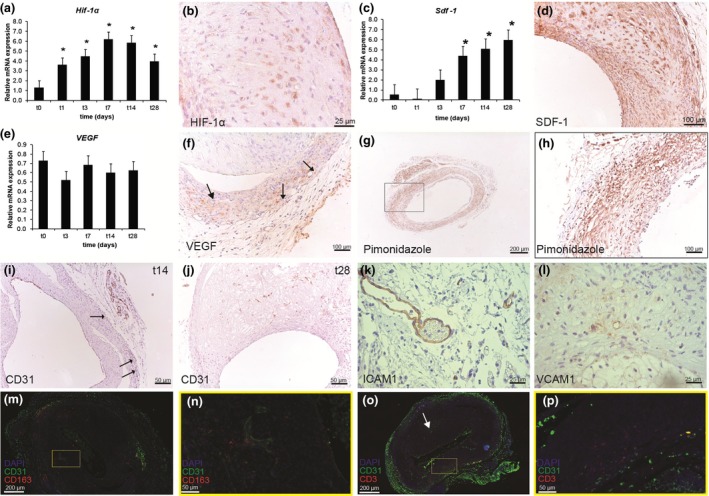
Hypoxia drives plaque angiogenesis. (a) Hypoxia‐inducible factor (Hif)‐1α mRNA regulation over time in vein grafts (t0‐t7 *n* = 3, t14 *n* = 4, t28 *n* = 5) in hypercholesterolaemic ApoE3*Leiden mice. (b) HIF‐1α protein is expressed by various cell types in vein grafts (t28). (c) Stromal cell‐derived factor 1 (Sdf‐1) mRNA regulation over time. (d) At day 28, SDF‐1 is expressed in all layers of the vein graft. (e) Vegf mRNA regulation over time. (f) VEGF could be seen in neovessels (arrows) in the vessel wall (t28). (g,h) Hypoxia (t28) could be detected with pimonidazole throughout the vein graft wall (*n* = 6 mice). (i) In a histological time course (*n* = 4 mice/time‐point), neovessels expressing CD31 could be detected from day 14 (t14) onwards. (j) At t28, neovessels could be detected throughout the entire vessel wall. Plaque neovessels show activation by the expression of intercellular adhesion molecule 1 (ICAM1) (k) and vascular cell adhesion molecule 1 (VCAM1) (l). CD163+ macrophages are found throughout the t28 vein graft lesion (m) and are clearly associated with neovessels (n). CD3+ T cells are found in the peri‐adventitial region of the vein grafts but not so much in other regions (white arrow) associated with neovessels (o,p) *P < 0.05.

In a time‐course experiment of murine vein grafts, the expression of *Hif1‐*α mRNA rapidly and significantly increased in vein grafts at all time‐points until day 28 (t28), when compared to native caval veins, with the highest level at t7, Fig. 2(a). *Hif1‐*α protein expression was clearly visible at t28, Fig. 2(b). *Sdf‐1* mRNA was significantly up‐regulated from t7 to t28 when compared to caval veins, Fig. 2(c). At the latter time‐point, *Sdf‐1* protein expression could be detected especially in SMCs, Fig. 2(d). Interestingly, while we could not detect an increase in *Vegf‐a* mRNA during the time course, Fig. 2(e), positive VEGF staining could be seen at t28, especially in plaque neovessels, Fig. 2(f). *In vivo,* we determined hypoxia by injecting the hypoxia probe pimonidazole (*n* = 6). Hypoxia was evident in all layers of the vein graft (t28), especially in macrophages scattered throughout the vein graft, Fig. 2(g,h).

A histological time course of vein grafts was used to study the timeframe in which the first plaque neovessels appear. From t14 (*n* = 4), the first plaque neovessels were detectable. These neovessels were primarily in the outer region of the vein grafts, suggesting sprouting from the vasa vasorum, Fig. 2(i). At t28, CD31+ plaque neovessels could be detected throughout all layers of the vein graft (*n* = 4), Fig. 2(j). The majority of these plaque neovessels have an activated endothelium, demonstrated by the expression of ICAM1 [Fig. 2(k)] and VCAM1, Fig. 2(l). Up‐regulation of ICAM1 and VCAM1 can lead to increased interactions with inflammatory cells. Therefore, 28‐day‐old vein grafts were stained with a combination of CD31 and CD163, an exclusive marker for neovessel‐associated macrophages 15 and CD31 and CD3+T cells. CD163+ macrophages can be abundantly found throughout the vein graft lesion [Fig. 2(m)] but mostly in close proximity of neovessels [Fig. 2(n)]. CD3+ T cells are mainly located in the peri‐adventitial region of the vein grafts which are highly vascularized [Fig. 2(o,p)]. However, CD3+ T cells are not specifically associated with neovessels in other areas within the vein graft lesion [white arrow, Fig. 2(o)].

### Perivascular VEGF increases plaque neovessel density

**Figure 3:**
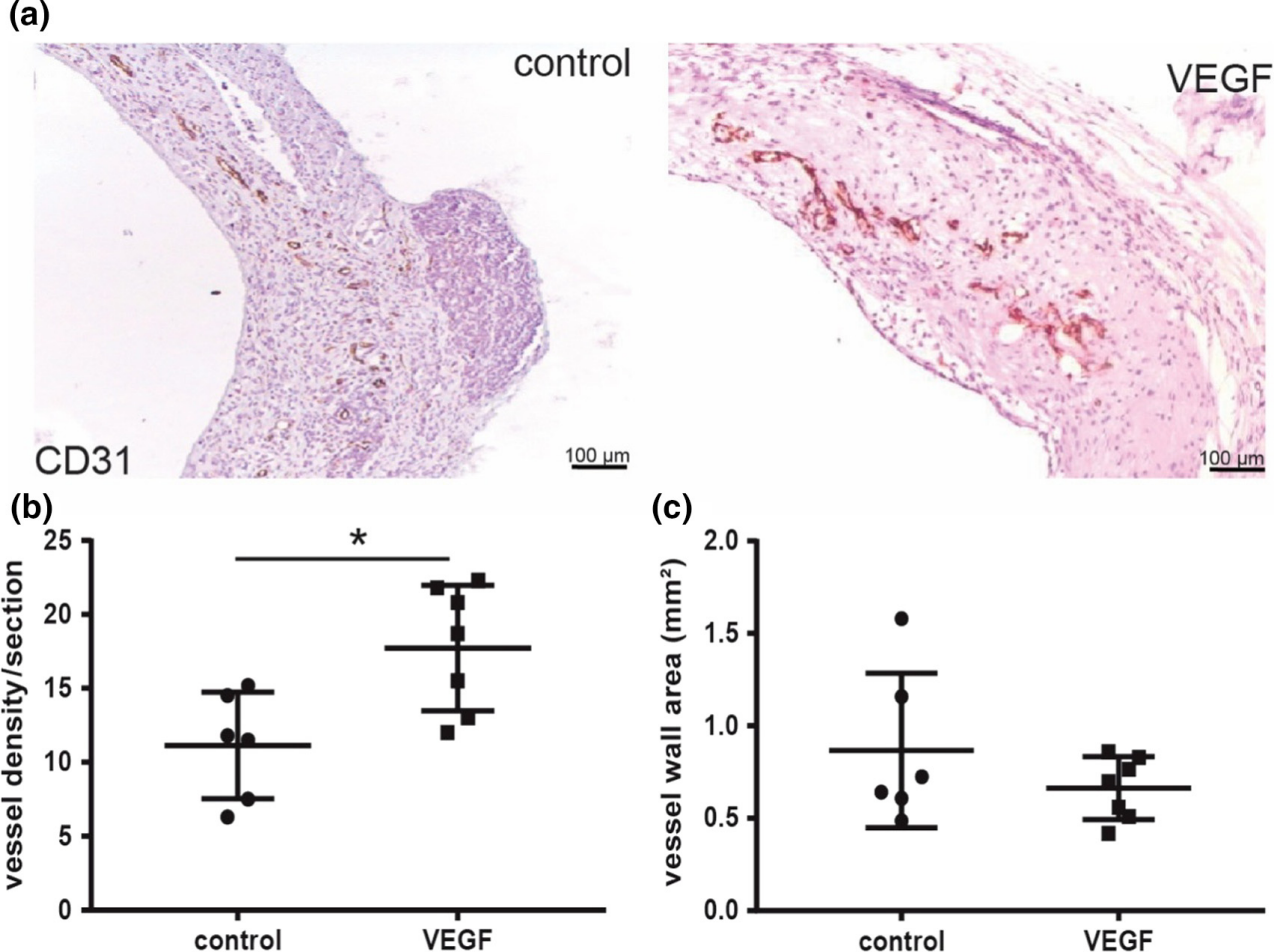
Perivascular VEGF increases plaque angiogenesis. VEGF was applied in pluronic gel (*n* = 7) or pluronic gel alone (*n* = 6) topically on the vein in ApoE3*Leiden mice. (a) CD31 staining of plaque neovessels in the control group (*n* = 6) and VEGF group (*n* = 7). (b) Quantification of the neovessel density at day 28. (c) Quantification of the vessel wall area. *P < 0.05.

To examine whether in the vein graft model we could target plaque angiogenesis, we applied a pluronic gel containing 250 ng VEGF in the perivascular region of the vein grafts, directly after surgery. Local treatment with VEGF did not affect cholesterol levels or bodyweight, Figure S1A,B. After 28 days, we observed an increase in the number of neovessels in the VEGF‐treated group compared to controls, Fig. 3(a). Quantification of the plaque neovessel density per section revealed a significant 60% increase in neovessels in the VEGF group (*P* = 0.014), Fig. 3(b). However, local application of VEGF did not result in a significant effect on the vessel wall area (*P* = 0.628), Fig. 3(c). In both the control group and VEGF‐treated group 1 out of 6 mice, intraplaque haemorrhage was observed.

### Angiopoietin expression is augmented in intraplaque haemorrhage regions

**Figure 4:**
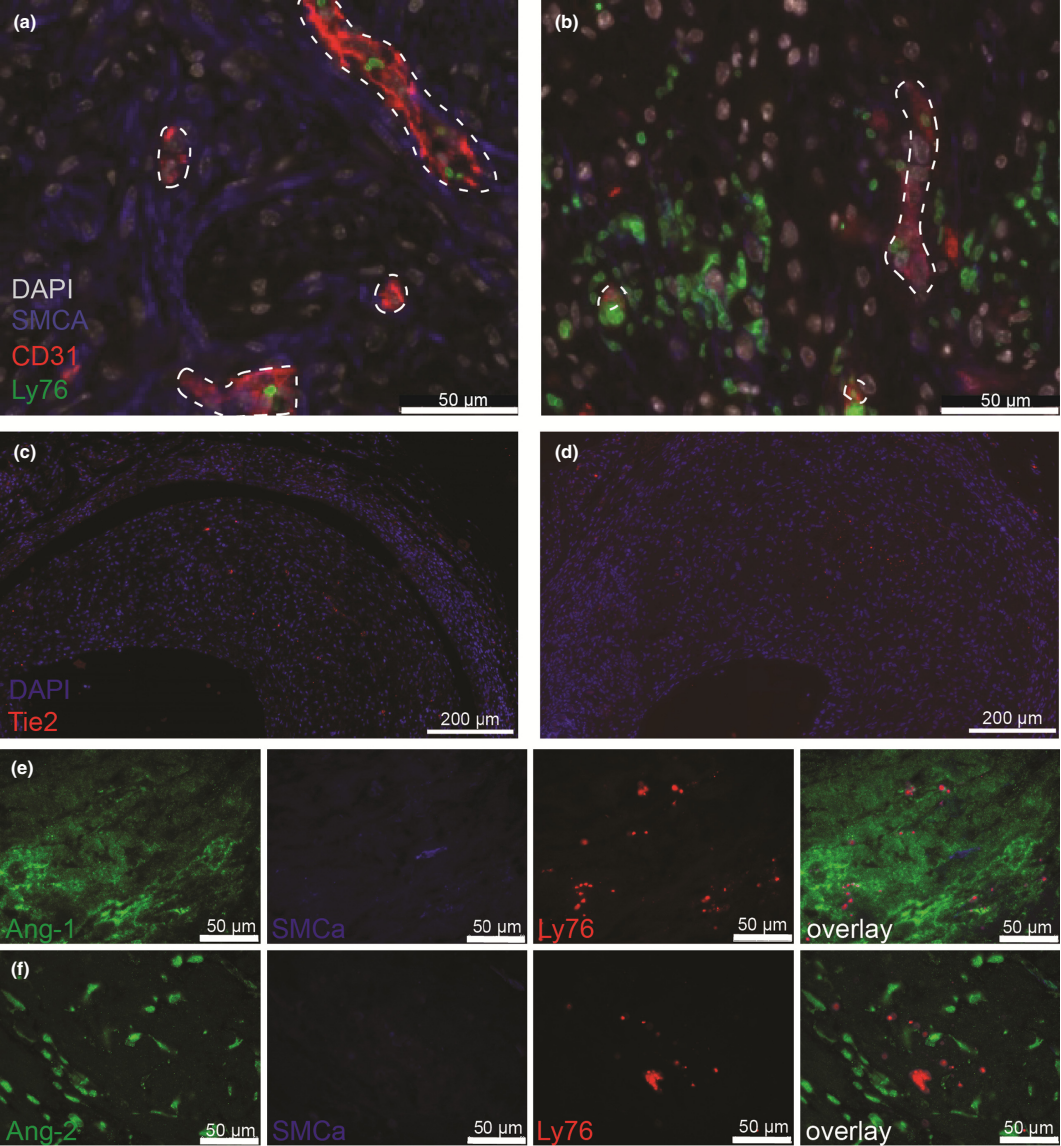
Angiopoietins–Tie2 expression in regions of intraplaque haemorrhage. (a) Staining of smooth muscle cell actin (SMCA), endothelial cells (CD31) and erythrocytes (Ly76) in plaque neovessels (dashed white lining) in lesion without (left panel) and with (right panel) intraplaque haemorrhage (IPH). (b) Staining of Tie2 in lesion without (left panel) and with (right panel) IPH. (c) Single staining and overlay of angiopoietin (Ang)‐1 (Ang‐1), SMCA and Ly76 in regions of IPH. (d) Single staining and overlay of Ang‐2, SMCA and Ly76 in regions of IPH.

Neovessels associated with intraplaque haemorrhage are characterized by reduced pericyte coverage. Mature neovessels are covered by SMC actin‐positive pericytes, Fig. 4(a). In regions of intraplaque haemorrhage (characterized by perivascular erythrocytes), neovessels were partly devoid of pericyte coverage, Fig. 4(b). Tie2, the main receptor of the angiopoietins, was found to be specifically expressed by endothelial cells of plaque neovessels. The expression of Tie2 did not differ between mature neovessels [Fig. 4(c)] or neovessels associated with intraplaque haemorrhage, Fig. 4(d). Increased staining of both Ang‐1 and Ang‐2 could be observed in areas of intraplaque haemorrhage. Ang‐1 was predominantly expressed in intraplaque haemorrhage regions, whereas no staining around mature neovessels could be observed, Fig. 4(e). Ang‐2 showed increased expression in lesions with intraplaque haemorrhage in contrast to regions of the lesions without intraplaque haemorrhage, Fig. 4(f).

### VEGFR2‐blocking antibodies inhibit intraplaque haemorrhage and erythrocyte extravasation

**Figure 5:**
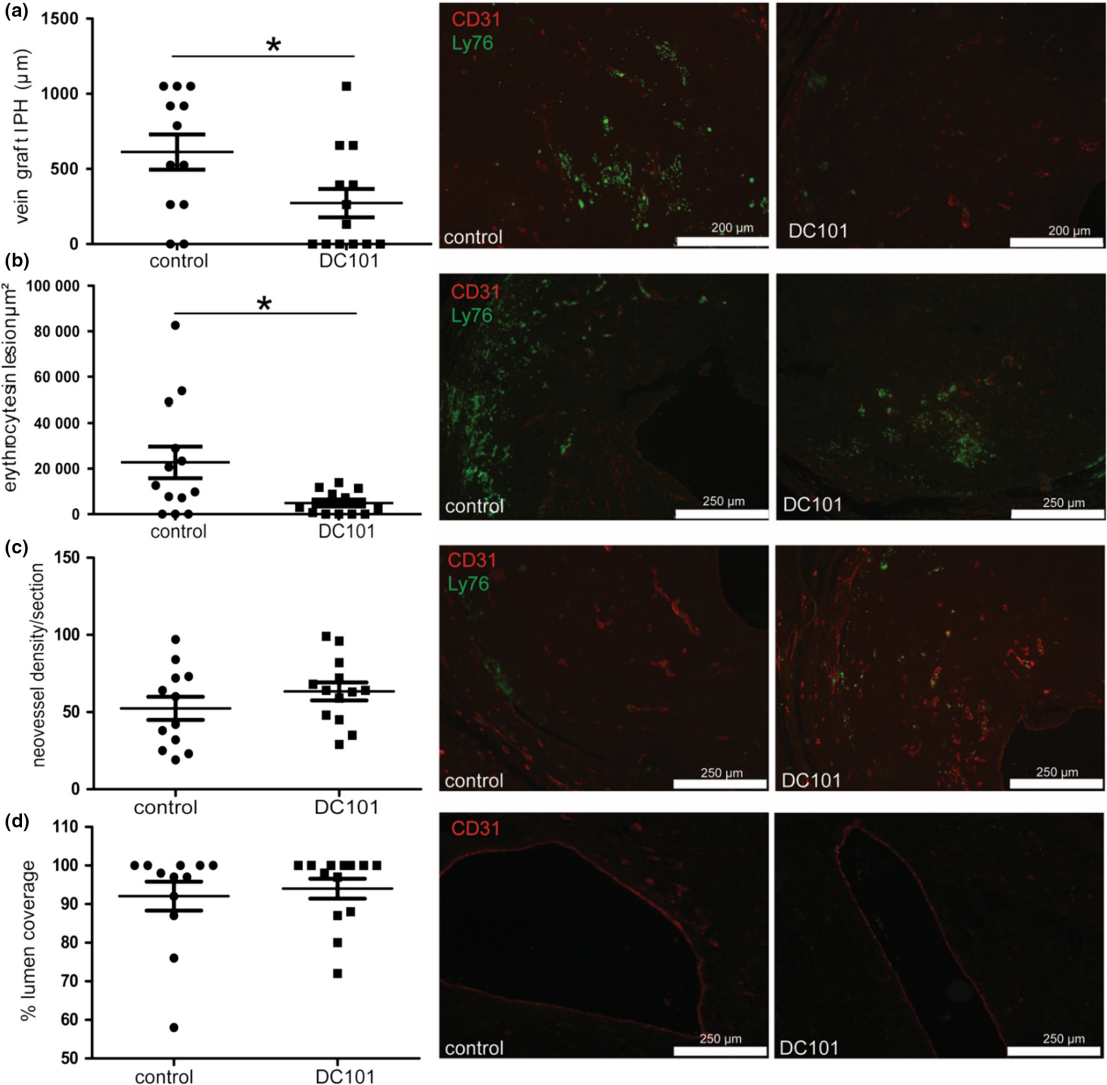
Intraplaque haemorrhage and erythrocyte extravasation after VEGFR2 blockade. Vein grafts in hypercholesterolaemic ApoE3*L mice treated with control IgG antibodies (10 mg kg^−1^) *n* = 12 and VEGFR2‐blocking antibodies (DC101, 10 mg kg^−1^) *n* = 14, 28 days after surgery. (a) Quantification of the vein graft length that displayed intraplaque haemorrhage. Representative examples of lesions with intraplaque haemorrhage. (b) Quantification of the area of the extravasated erythrocytes and representative images of lesions with extravasated erythrocytes. (c) Quantification of the density of neovessels in vein grafts expressed as the number of neovessel per section (10–12 sections/mouse) (d). Quantification of endothelial cell coverage of the lumen expressed as % coverage. *P < 0.05, L, lumen.

To interfere in the process of vessel integrity, we treated ApoE3*Leiden receiving a vein graft with the VEGFR2‐blocking antibody (DC101). Treatment with DC101 did not change cholesterol levels or bodyweight in comparison with the control group, Figure S1C,D. Intraplaque haemorrhage was less frequently observed in mice treated with DC101 (7 out of 14 mice, 50%) in comparison with control animals (10 out of 12 mice, 83%). In the DC101 group, a smaller segment of the vein grafts (242 μm, 26% of the vein graft length) was affected by intraplaque haemorrhage in comparison with the control group (620 μm, 59% of the vein graft length, *P* = 0.037), Fig. 5(a). In addition, 80% less extravasated erythrocytes were observed in the DC101 group than in the control group (*P* = 0.049), Fig. 5(b). These extravasated erythrocytes were predominantly observed in the regions near the adventitia and in the mid‐portion of the vein graft lesions, Fig. 5(b).

### Neovessel density is not reduced by VEGFR2‐blocking antibodies *in vivo*

The anti‐angiogenic effect of suppression of VEGF‐signalling *in vivo* was analysed by quantifying the neovessel density in the vein graft lesions. In the DC101 group, an average of 63 ± 25 neovessels per vein graft section was observed, whereas in the control IgG‐treated group, 52 ± 19 neovessels per vein graft section were found (*P* = 0.327), Fig. 5(c). The vein graft model is characterized by the denudation of the luminal endothelium in the early days after engraftment, which is restored later in time 5. In the both DC101 group and control group, the endothelium was completely restored at 28 days after surgery (*P* = 0.639), Fig. 5(d).

### DC101 prevents vein graft thickening and results in a more stable lesion composition

**Figure 6:**
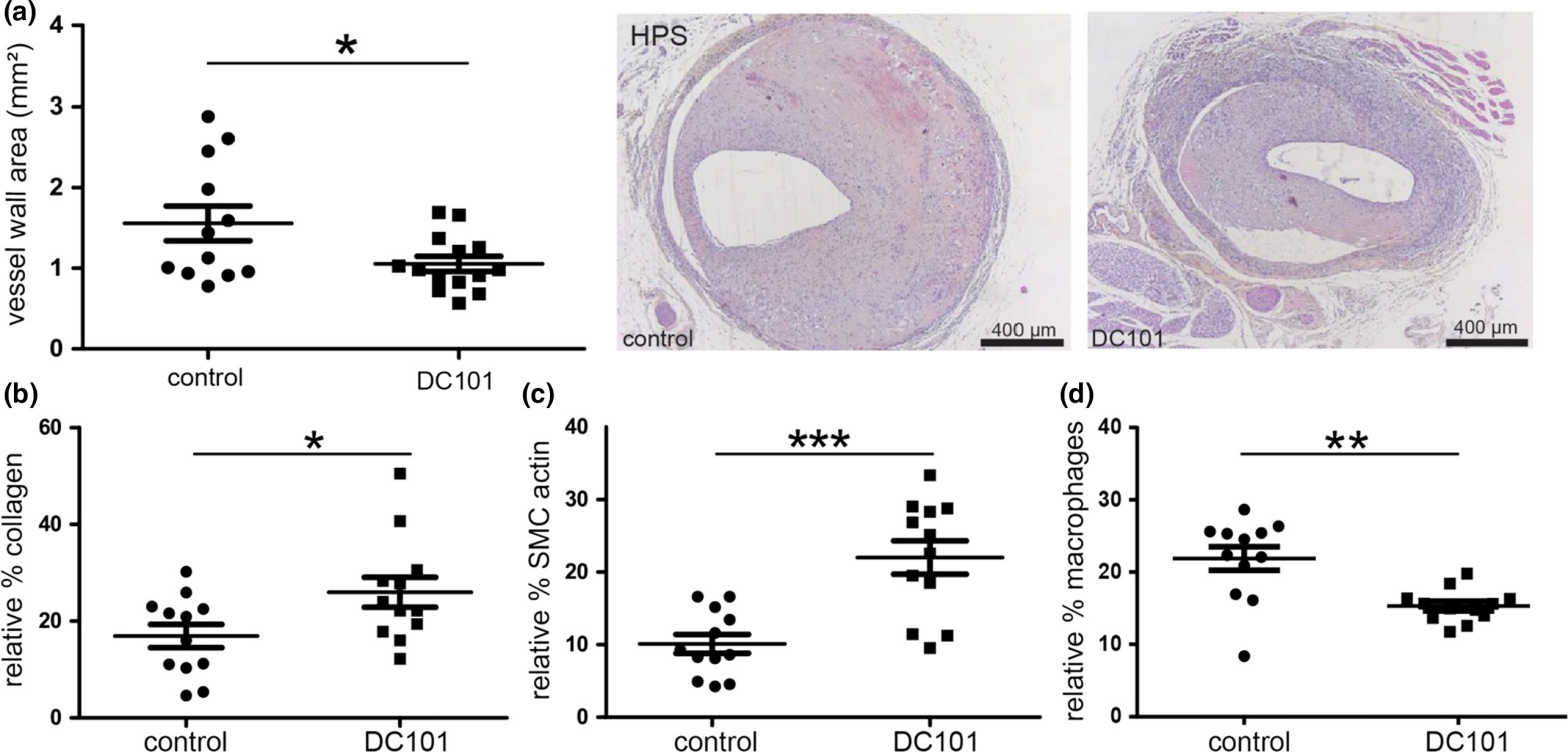
Quantitative measurements of vein graft area and lesion composition. (a) Quantitative measurements of vein graft thickening and representative cross sections of vein grafts in hypercholesterolaemic ApoE3*L mice treated with control IgG antibodies (10 mg kg^−1^) *n* = 12 and VEGFR2‐blocking antibodies (DC101, 10 mg kg^−1^) *n* = 14, 28 days after surgery (Haematoxylin–Phloxine–Saffron staining). (b) Relative percentage collagen, (c) relative percentage smooth muscle cell actin, (d) relative percentage macrophages. *P < 0.05, **P < 0.01, ***P < 0.005.

VEGFR2 blockade resulted in a reduction in the lesion size compared to the control group, Fig. 6(a). Quantification of these lesions showed that the DC101‐treated group had a significant reduction of 32% in vein graft thickening compared to the control IgG‐treated group (*P* = 0.044), Fig. 6(a). A decrease in outward remodeling as measured by the total vessel area was detected in the DC101‐treated group (33%, *P* = 0.05), Figure S2A. The luminal area, however, was not significantly affected by DC101 treatment (*P* = 0.369), Figure S2B. Next, the effect of DC101 treatment on vein graft lesion composition was assessed. In the DC101 group, an increased collagen content was observed in comparison with the control group (46%, *P* = 0.066), Figure S2C. When corrected for the differences in vein graft thickening, the relative percentage of collagen was significantly increased in the DC101‐treated group (54% *P* = 0.047), Fig. 6(b). In addition, a substantial increase in the SMCA area was observed (118% *P* = 0.003) in the DC101 group as well as a significant increase in the percentage of SMCA (123% *P* = 0.0005), Fig. 6(c) and Figure S2D. Plaque macrophages were significantly reduced after DC101 treatment with 30% (*P* = 0.001), Fig. 6(d), whereas the total macrophage area was reduced by 42% (*P* = 0.018), Figure S2E.

### VEGFR2 blockade stimulates expression of genes associated with a more mature neovessel phenotype

**Figure 7:**
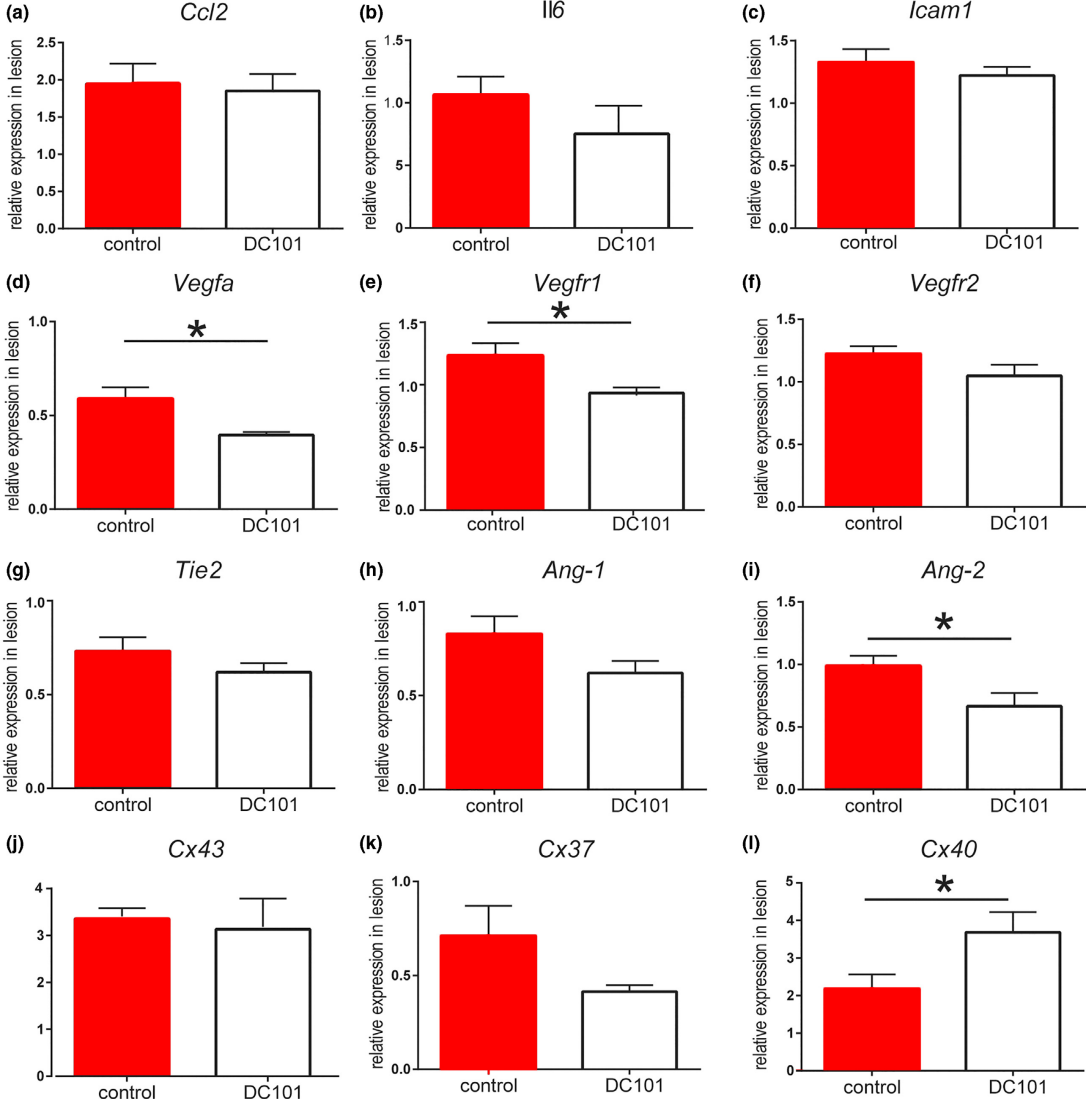
Gene expression in vein grafts. Total wall gene expression was measured in vein grafts of control and VEGFR2‐blocking antibodies that treated mice (*n* = 6/group). (a) Inflammation‐associated genes. (b) VEGF and VEGF receptor genes. (c) Tie2 and angiopoietins. (d) Connexins. *P < 0.05.

To investigate the local inflammatory response, we measured the gene expression levels of pro‐inflammatory genes *Ccl2, Il6* and *Icam1* in the vein grafts; no differences in expression levels could be detected between the groups, Fig. 7(a–c). Also, the expression of VEGF/VEGFR mRNA in the vein graft wall was analysed. Interestingly, the expression of both *Vegfa* [Fig. 7(d)] and *Vegfr1* [Fig. 7(e)] was significantly reduced upon DC101 treatment [24% (*P* = 0.014) and 32% (*P* = 0.048), respectively], whereas the expression of *Vegfr2* was not affected, Fig. 7(f). Furthermore, the angiopoietin receptor *Tie2* [Fig. 7(g)] was not differently expressed between the groups, nor was the vessel stabilizing factor *Ang1*, Fig. 7(h). The vessel destabilizing factor *Ang2* was significantly decreased (*P* = 0.039) after DC101 treatment, Fig. 7(i). As a measure for proper endothelial function, we measured Connexin (*Cx*43, *Cx37* and *Cx40*) expression. DC101 treatment showed no effect on *Cx43* [Fig. 7(j)] and *Cx37* [Fig. 7(k)] expression levels, but remarkably, significantly increased (*P* = 0.047) levels of *Cx40* were observed pointing towards increased interendothelial cell connections, Fig. 7(l).

### VEGFR2‐blocking antibodies induce concentration‐dependent vessel maturation

**Figure 8:**
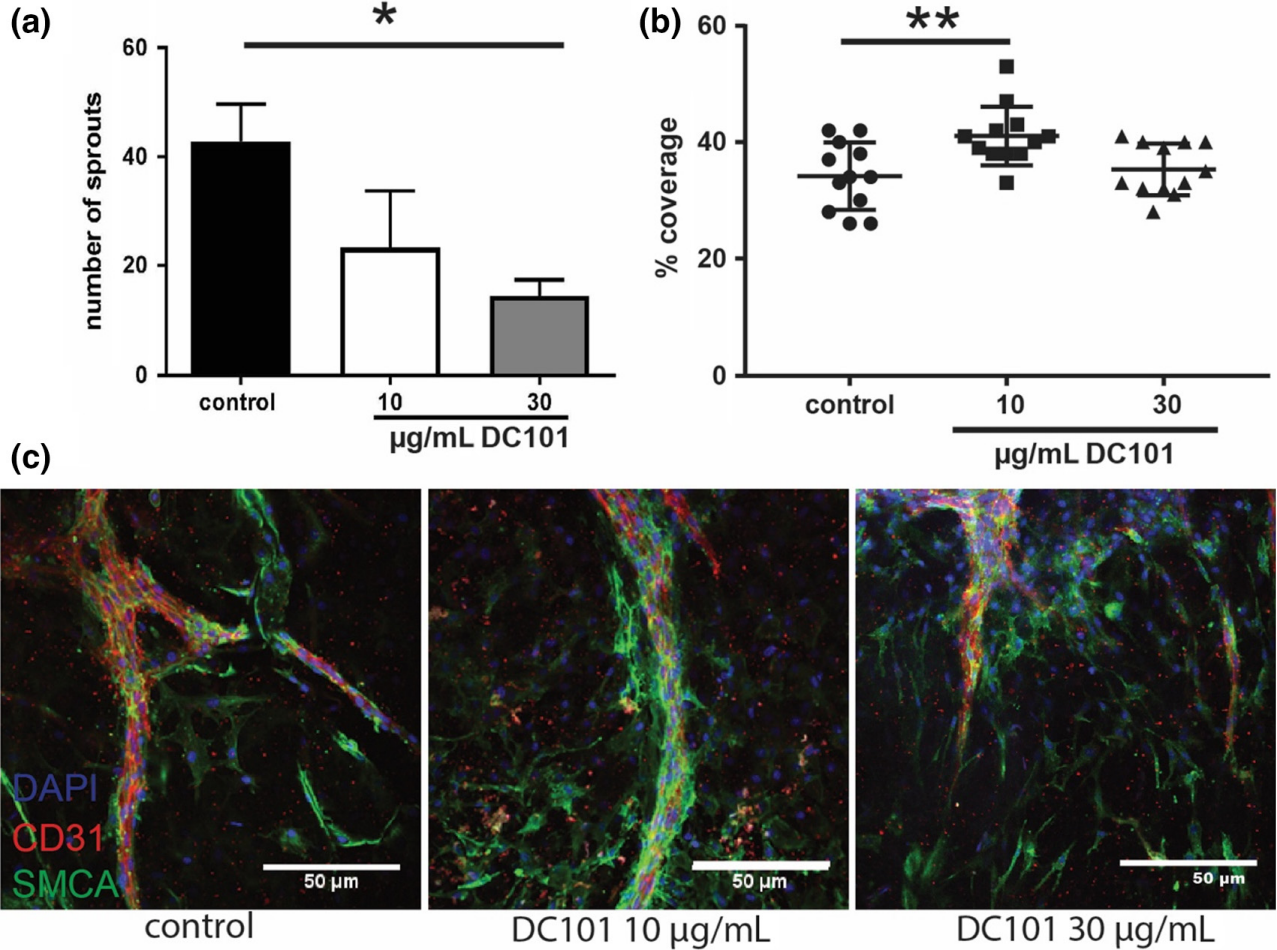
VEGFR2 blockade in aortic ring assay. Representative experiment of three separate experiments using three mice/condition and four rings per mouse. Quantification of the number of sprouts (a) and the % coverage of SMCs over the CD31^+^ sprouts (b). (c) Typical examples of sprouts in the control and DC101‐treated groups. *P < 0.05, **P < 0.01.

The effects of VEGFR2 blockade on vessel maturation were further studied in an aortic ring assay. Of the two concentrations DC101 (10 and 30 μg mL^−1^) tested, only the highest concentration resulted in a significant reduction (66% *P* = 0.003) in sprout formation when compared to no treatment, Fig. 8(a). The pericyte coverage of the sprout in the 30 μg mL^−1^ DC101 group was not significantly different than the control. Interestingly, the 10 μg mL^−1^ DC101 concentration induced a significant increase in SMCA+ pericyte coverage of the CD31+ sprouts (20%, *P* = 0.005), Fig. 8(b,c).

## Discussion

Immature intraplaque neovessels have been characterized as the main contributors to intraplaque haemorrhage. Intraplaque haemorrhage occurs in native atherosclerosis but also in accelerated atherosclerosis after vein grafting or stenting 4. However, most of the evidence is descriptive in nature 9, 13. In the present study, we used an intervention to show that VEGFR2 blockade reduces intraplaque haemorrhage and increases plaque stability by enhancing neovessels maturation in vein graft atherosclerosis.

We observed that neovessels in human carotid and vein graft specimen are associated with VEGF/VEGFR2 and angiopoietins. In both types of atherosclerotic lesions, numerous regions with intraplaque haemorrhage and leaky vessels were observed. We demonstrated that plaque neovessels in the vein grafts originate primarily from the adventitia. This is also the general idea for native atherosclerotic lesion; however, luminal angiogenesis cannot be excluded 1. VEGFR2 is involved in this process as the main receptor. VEGFR2 is involved in tip‐cell‐stalk‐cell differentiation in the early phase of angiogenesis and mediates the permeability‐enhancing effects of VEGF in adult endothelial cells as well as neovessel maturation 16. We have previously shown that the majority of plaque neovessels in vein grafts express a basement membrane and that pericyte coverage is heterogeneous 23. Here, we demonstrate that incomplete pericyte coverage of murine plaque neovessels is angiopoietin‐related. Incomplete pericyte coverage in regions of intraplaque haemorrhage is also observed in human instable atherosclerotic plaques 11.

A modest induction in Vegf mRNA expression between 3 and 7 days but no further regulation between other time‐points was observed. Interestingly, Hamdan *et al*. 27 showed comparable absent induction of Vegf mRNA in a canine vein graft model between native vein and 4 weeks after surgery, but did see a significant induction after 48 h. It seems that Vegf mRNA expression is only induced for a short period and is not the main driver of the remodelling response after vein graft surgery. This early induction of Vegf mRNA expression can be a result of the hypoxic period during surgery.

Atherosclerotic plaque angiogenesis can be manipulated as we show here by intervening in the VEGF pathway: locally applied VEGF‐enhanced neovessel density. We found that low concentration of VEGFR2‐blocking antibodies induced pericyte coverage in the aortic ring assay. This is comparable to the observation of increased pericyte coverage in murine and human tumours after VEGF signal blockade 28. Blockade of VEGFR2 has been shown to facilitate the recruitment of pericytes to tumour vessels by enhancing Ang‐1 expression and increasing perivascular matrix metalloproteases activity 29. Ang‐1 decreases endothelial cell permeability and increases vascular stabilization via enhancing endothelial cell interactions with the surrounding matrix and recruitment of pericytes to growing blood vessels. Ang‐2 functions as a competitive Ang‐1 antagonist in a VEGF‐dependent manner and mediates angiogenic sprouting and vascular regression 19. This concurs with our finding that in regions of intraplaque haemorrhage, the expression of both Ang‐1 and Ang‐2 is increased. VEGFR2 blockade by DC101 treatment reduced intraplaque haemorrhage, reduced *Ang‐2* expression and improving gap junctions as shown by the increased *Cx40* expression, pointing towards more mature neovessels. Post *et al*. 30 showed that in plaques with high neovessel density, the local balance between Ang‐1 and Ang‐2 is in favour of Ang‐2. Unfortunately, vascular maturation and intraplaque haemorrhage were not studied in this context.

Recently, it was shown that treatment with axitinib (inhibitor of VEGFR1, 2 and 3) attenuated plaque angiogenesis 31. Treating vein grafts with VEGFR2‐blocking antibodies *in vivo* did not result in a reduction in neovessel density in comparison with control IgG‐treated animals. Interestingly in a model for breast cancer, tumour vascular density was also not affected with this dose (10 mg kg^−1^ DC101) but was significantly decreased with a four times higher dose 32. Furthermore, these authors observed that low‐dose but not high‐dose VEGFR2‐blocking antibodies treatment resulted in improved vascular maturation. In the aortic ring assay, we observed that the high‐dose DC101 resulted in reduced sprouting, whereas the low dose did not reduce sprouting but did increased pericyte coverage.

VEGF is known to induce re‐endothelialization and has been shown to inhibit intimal hyperplasia after vascular injury 33. Application of VEGF directly after surgery in a rabbit vein graft model showed attenuation of the vessel wall size 34. We show that local delivery of VEGF directly after surgery results in a nonsignificant trend towards reduction in intimal hyperplasia, whereas blockade of VEGFR2 resulted in significant attenuation of lesion growth. In the VEGFR2 blockade experiment, treatment with DC101 was started at day 14 after surgery to specifically study the effects on plaque neovessel formation which starts from this time‐point on as demonstrated in Fig. 2(i). An important mechanism of action of VEGF is enhancing the re‐endothelialization of the luminal endothelium which occurs primarily in the early period after surgery 35. The late treatment with DC101 does not interfere with the re‐endothelialization process. This was confirmed by the observation that at sacrifice (t = 28 days) both the control and DC101 group showed full luminal endothelial coverage. Vein graft lesion formation is largely driven by inflammation 36. The positive effect of the VEGFR2 blockade on this process most likely overrules the VEGF‐induced attenuation of lesion growth.

It has been shown that VEGFR2 activation can activate and degrade VE‐cadherin resulting in vascular permeability 37. Guo *et al*. 15 showed that CD163+ macrophages promote endothelial permeability via VEGF/VEGFR2 interaction with VE‐cadherin. These CD163+ macrophages are clearly present, localized in areas of plaque neovascularization, in the murine vein grafts (Fig. 2). Blockade of VEGFR2 could reverse the VE‐cadherin induced vascular permeability and induce the observed plaque neovessel maturation and reduced intraplaque haemorrhage.

Phagocytosis of intraplaque erythrocytes and erythrocyte‐derived cholesterol by macrophages results in lipid core and plaque expansion, and promotion of plaque instability 12, 38. Systematic VEGFR2 blockade led to a reduction in intraplaque haemorrhage, lesion size and a reduction in lesion macrophages. Binding of VEGF to VEGFR2 can result in NF‐κB‐induced activation of VCAM‐1 and ICAM‐1 leading to increased adherence of leucocytes 39. In various experimental models, inhibition of vascular leakage and NF‐κB‐dependent macrophage influx by DC101 was demonstrated 40, 41. Although at t28 no effect on inflammatory gene expression could be seen in the vein grafts, blockade of the binding of VEGF to VEGFR2 inhibited macrophage influx and subsequent effects on plaque composition including increased collagen and smooth muscle cell content. The NF‐κB signalling cascade is an obvious route, since NF‐κB‐induced inflammation has been previously reported to be a critical pathway to stimulate macrophage influx and plaque instability in vein grafts 24, 36, 42.

In this study, we used a vein graft model in hypercholesterolaemic mice to study the role of plaque neovessel maturation. This model shows large atherosclerotic lesions with abundant plaque angiogenesis 23. Vein graft atherosclerosis differs from native atherosclerosis since the onset (surgery) is acute with endothelial denudation and hypoxia resulting in the accelerated form. The lesions formed are concentric and highly dispersed with inflammatory cells and foam cells 36. Local processes regarding plaque neovessel maturation in vein grafts show high similarities with native atherosclerosis as demonstrated in Fig. 1. The findings in this study can be, with cause, extrapolated to other cardiovascular diseases.

In summary, VEGFR2‐blocking antibodies inhibit intraplaque haemorrhage and erythrocyte extravasation, resulting in more stable plaque neovascularization, decreased lesion development and increased plaque stabilization in a vein graft model, due to the maturation of the plaque neovessels. Our study indicates that vascular maturation (and more specifically VEGFR2) stands as an attractive target to stabilize atherosclerotic (vein graft) disease.

## Author contributions

MdV, MJG and PQ designed the experiments and interpreted data. MdV, LP, EP, AS, LG and AF performed experiments and analysed data. JH, JWJ, CKO and RV provided intellectual contributions throughout the project. MdV and PQ wrote the manuscript and were responsible for the overall supervision of the manuscript. All authors discussed the results and commented on the manuscript.

## Funding

This work was supported by a grant from the European Union, MSCA joint doctoral project [675527], National Institutes of Health [1R01HL133500] and American Heart Association Grant‐in‐Aid [16GRNT27090006].

## Conflict of interest

None declared.

## Supporting information


**Table S1.** Patient characteristics saphenous vein grafts.
**Figure S1.** Bodyweight and cholesterol levels.
**Figure S2.** Vein graft morphometry.
