# Supplementary material for: Blockade of vascular endothelial growth factor receptor 2 inhibits intraplaque haemorrhage by normalization of plaque neovessels
Source: J Intern Med. 2018 Sep 7;285(1):59–74. doi: 10.1111/joim.12821 (PMC6334526; doi:10.1111/joim.12821)
Supplement: Supplementary file 1 — Table S1. Patient characteristics saphenous vein grafts. Figure S1. Bodyweight and cholesterol levels. Figure S2. Vein graft morphometry. [file JOIM-285-59-s001.docx]

Supplemental information

STable 1. **Patient characteristics saphenous vein grafts**

| Age | Sex | % stenosis | %stenosis  bypass | Stage | Smoker | Diabetes I | Diabetes II | HLP | Previous  MI | Heart  Failure | CAD | Renal failure | Hypertension |
| --- | --- | --- | --- | --- | --- | --- | --- | --- | --- | --- | --- | --- | --- |
| 58 | male | 60 | 30 | Intermediate | no | no | no | no | yes | no | yes | no | yes |
| 54 | male | 80-90 | 80 | Late | no | no | no | no | no | no | no | no | no |
| 37 | male | 60 | 75 | Late | no | no | no | no | no | no | no | no | no |
| 58 | male | 60 | 75 | Late | no | no | no | no | no | no | no | no | no |
| 92 | male | 40 | 40 | Intermediate | no | no | no | no | no | no | yes | no | no |
| 76 | male | 70 | 70 | Late | no | no | no | no | no | no | yes | no | no |
| 62 | male | 50 | 70 | Late | no | no | yes | yes | no | no | yes | no | yes |
| 62 | male | 50 | 25 | Early | yes | no | no | no | no | no | yes | no | yes |
| 62 | male | 80 | 70 | Late | yes | no | no | no | no | no | yes | no | yes |
| 70 | female | 60 | 40 | Intermediate | no | no | no | no | no | no | no | no | no |
| 70 | female | 30 | 30 | Early | no | no | no | no | no | no | no | no | no |
| 76 | male | 70 | 35 | Intermediate | no | no | no | no | no | no | no | no | no |

*Abbreviations: HLP – hyperlipoproteinemia; MI – myocardial infarction; CAD – coronary artery disease. I n red photographs in figure 1, panel 1.*


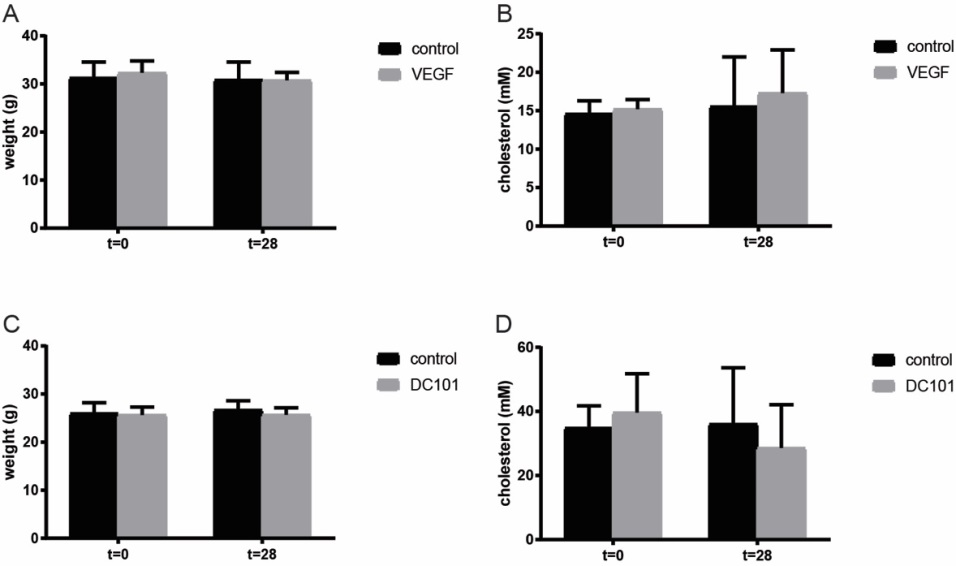


Figure S1. **Bodyweight and cholesterol levels**. (**A**). Bodyweight before (t=0) and 28 days after surgery (t=28) of VEGF treated and control mice. (**B**). Plasma cholesterol levels before (t=0) and 28 days after surgery (t=28) of VEGF treated and control mice. (**C**). Bodyweight before (t=0) and 28 days after surgery (t=28) of VEGFR2-blocking antibodies (DC101) treated and control mice. (**D**). Plasma cholesterol levels before (t=0) and 28 days after surgery (t=28) of DC101 treated and control mice.


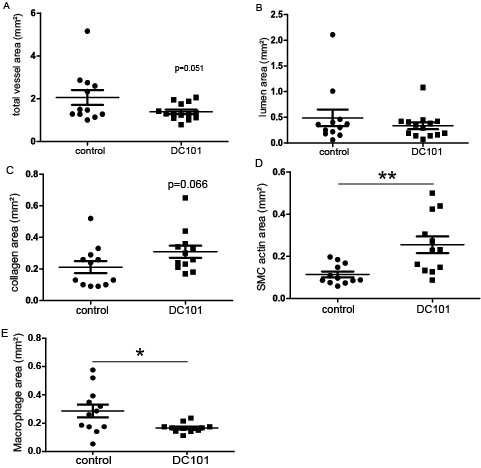


Figure S2. **Vein graft morphometry**. Quantitative measurements of vein graft area and lesion composition in vein grafts in hypercholesterolaemic ApoE3*L mice treated with control IgG antibodies (10 mg/kg) n=12 and VEGFR2-blocking antibodies (DC101, 10 mg/kg) n=14, 28 days after surgery. Quantitative measurements of (A) total vessel area, (B) luminal area, (C) collagen area, (D) smooth muscle cell (SMCA) area, (E)macrophage content. * p<0.05, **p<0.01.
